# Adverse Drug Reactions in Children—A Systematic Review

**DOI:** 10.1371/journal.pone.0024061

**Published:** 2012-03-05

**Authors:** Rebecca Mary Diane Smyth, Elizabeth Gargon, Jamie Kirkham, Lynne Cresswell, Su Golder, Rosalind Smyth, Paula Williamson

**Affiliations:** 1 School of Nursing, Midwifery and Social Work, University of Manchester, Manchester, England, United Kingdom; 2 Department of Biostatistics, University of Liverpool, Liverpool, England, United Kingdom; 3 Centre for Reviews and Dissemination, University of York, York, England, United Kingdom; 4 Department of Women's and Children's Health, University of Liverpool, Liverpool, England, United Kingdom; Yale University School of Medicine, United States of America

## Abstract

**Background:**

Adverse drug reactions in children are an important public health problem. We have undertaken a systematic review of observational studies in children in three settings: causing admission to hospital, occurring during hospital stay and occurring in the community. We were particularly interested in understanding how ADRs might be better detected, assessed and avoided.

**Methods and Findings:**

We searched nineteen electronic databases using a comprehensive search strategy. In total, 102 studies were included. The primary outcome was any clinical event described as an adverse drug reaction to one or more drugs. Additional information relating to the ADR was collected: associated drug classification; clinical presentation; associated risk factors; methods used for assessing causality, severity, and avoidability. Seventy one percent (72/102) of studies assessed causality, and thirty four percent (34/102) performed a severity assessment. Only nineteen studies (19%) assessed avoidability. Incidence rates for ADRs causing hospital admission ranged from 0.4% to 10.3% of all children (pooled estimate of 2.9% (2.6%, 3.1%)) and from 0.6% to 16.8% of all children exposed to a drug during hospital stay. Anti-infectives and anti-epileptics were the most frequently reported therapeutic class associated with ADRs in children admitted to hospital (17 studies; 12 studies respectively) and children in hospital (24 studies; 14 studies respectively), while anti-infectives and non-steroidal anti-inflammatory drugs (NSAIDs) were frequently reported as associated with ADRs in outpatient children (13 studies; 6 studies respectively). Fourteen studies reported rates ranging from 7%–98% of ADRs being either definitely/possibly avoidable.

**Conclusions:**

There is extensive literature which investigates ADRs in children. Although these studies provide estimates of incidence in different settings and some indication of the therapeutic classes most frequently associated with ADRs, further work is needed to address how such ADRs may be prevented.

## Introduction

Adverse drug reactions (ADR) are a major health problem to the individual as well as for society [1]. The World Health Organisation's definition of an ADR is “a response to a drug which is noxious, and unintended, and which occurs at doses normally used in man for prophylaxis, diagnosis or therapy of disease, or for the modification of physiological function” [2]. The frequent occurrence' of ADRs in children has been reported in three previous systematic reviews of observational studies covering the period from 1966 to 2010 [3], [4], [5]. The reviews provided estimates of ADR rates causing hospital admission, in hospitalised children and in outpatient children and demonstrated that ADRs in hospitalised children are a considerable problem. Two of the reviews [4], [5] provide data on the clinical presentation of the ADR and the drugs involved. In addition, the more recent review [5] provides information on the methods and persons involved in identifying ADRs.

There are however, a number of limitations to the previous reviews. Each review [3], [4], [5] applied a search strategy, using a limited number of keywords to just two electronic bibliographic databases - MEDLINE and EMBASE. Importantly, as a consequence, relevant studies may have been excluded. In addition, the reviews excluded studies that included adults as well as children, thus reducing the number of eligible studies, and the more recent reviews excluded studies that evaluated adverse drug events (medication errors as well as ADRs).

These reviews do not provide information about the drugs involved in ADRs or about which methods were used for detecting, or assessing the causality and subsequent of an ADR [6]. Establishing the relationship between the drug and suspected reaction is fundamental to drug safety and being able to determine the avoidability [7] of an ADR in order to try to prevent its future occurrence is crucial to reducing the burden of ADRs.

We therefore undertook this systematic review to provide a more comprehensive assessment of all relevant studies and to understanding how ADRs might be better detected, assessed and avoided.

## Methods

### Criteria for considering studies for this review

#### Included studies

Observational studies that estimate the incidence of ADRs including retrospective and prospective cohort studies of children.

#### Excluded studies

Studies which focus on ADRs in relation to a specific drug (e.g. antibiotics or carbamazepine), clinical condition (e.g. epilepsy, asthma) or specific clinical presentations of ADRs (anaphylaxis); case control studies; those carried out exclusively on a neonatal intensive care unit; studies reporting medication errors, therapeutic failures, non-compliance, accidental and intentional poisoning and drug abuse.

#### Participants

Children as defined by the original study authors.

Studies included three defined populations: 1) children admitted to hospital, 2) children in hospital and 3) children within the community.

#### Interventions

Exposure to any systemic or topical medicinal product including herbals and aromatherapy, as defined by researchers.

#### Types of outcome measure

Any clinical event described as an adverse drug reaction or non-avoidable adverse drug event to an individual or group of drugs.

### Search methods for identification of studies

A range of electronic bibliographic databases were searched (Table 1) using a search strategy of text words and indexing terms (Table 2). In addition, we examined references in relevant studies and those cited by previous systematic reviews. Contact with experts was made to identify other potentially relevant published and unpublished studies. We did not apply language restrictions to the search.

**Table 1:** Databases searched.

Database	
MEDLINE via OVID	1950 to October 2010
EMBASE via NHS Evidence Health Information Resource	1980 to October 2010
CINAHL via NHS Evidence Health Information Resources	1981 to October 2010
Science Citation Index (SCI) via ISI Web of Knowledge	1990 to October 2010
Biological Abstracts via OVID	1926 to October 2010
International Pharmaceutical Abstracts (IPA) via OVID	1970 to October 2010
Toxicology Literature Online – via USA National Library of Medicine	searched October 2010
Iowa Drug Information Service (IDIS) via University of Iowa	1966 to October 2010
Allied and Complimentary Medicine Database (AMED) via OVID	1985 to October 2010
General Practice Research Database via http://www.gprd.com/home/	1987 to October 2010
Database of Systematic Reviews *(The Cochrane Library)* via http://www.thecochranelibrary.com	searched October 2010
Database of Abstracts of Reviews of Effects (DARE) via University of York	searched October 2010
Health Technology Assessment Programme via http://www.hta.ac.uk/index.shtml	searched October 2010
National Institute of Health via http://www.nih.gov/	searched October 2010
European Medicines Agency via http://www.ema.europa.eu/ema	searched October 2010
US Food and Drug Administration via http://www.fda.gov/	searched October 2010
Clinicaltrials.gov via http://clinicaltrials.gov/	searched October 2010
Agency for Health and Research Quality via http://www.ahrq.gov/	searched October 2010
Incidence and Prevalence via http://www.dialog.com/proquestdialog	searched November 2010

**Table 2:** MEDLINE search strategy.

**1^st^ Concept - general terms used to describe the participants - infants and children.**1. exp Child/2. exp Adolescent/3. (young adj (person$ or people or adult$ or individual$ or women or woman or men or man)).ti,ab.4. (child$ or adolescen$ or kid or kids or youth$ or youngster$ or minor or minors or teen$ or juvenile$ or student$ or pupil$ or boy$ or girl$).ti,ab.5. exp Students/6. Puberty/7. Pediatrics/8. (infan$ or newborn$ or new born$ or baby$ or babies or child$ or schoolchild$ or kid or kids or toddler$ or adoles$ or teen$ or boy$ or girl$ or minor$ or juvenil$ or youth$ or kindergar$ or nurser$ or puber$ or prepuber$ or pre puber$ or pubescen$ or prepubescen$ or pre pubescen$ or pediatric$ or paediatric$ or schoolage$).ti,ab.
**2^nd^ Concept including terms relating to adverse drug reactions**9. side effect$.ti,ab.10. (drug induced or drug related or drug safety).ti,ab.11. tolerability.ti,ab.12. toxicity.ti,ab.13. Harm$.ti,ab.14. adrs.ti,ab.15. (adverse adj2 (effect or effects or reaction or reactions or event or events or outcome or outcomes)).ti,ab.16. (toxic adj3 (effect$ or reaction$ or event$ or outcome$)).ti,ab.17. exp product surveillance, postmarketing/ or exp adverse drug reaction reporting systems/ or exp drug toxicity/ or exp abnormalities, drug induced/ or exp drug hypersensitivity/
**3^rd^ Concept – terms relating to the occurrence of ADRs**18. incidence/ or prevalence/19. (incidence$ or prevalence$ or occurrence or admission$ or admitted or visit$ or hospitalisation or hospitalised or hospitalization or hospitalized).ti,ab.
**4^th^ Concept - terms that encompass the intervention**20. (drug$ or pharmaceutical$ or medicin$).ti,ab.21. Pharmaceutical Preparations/22. (herbal$ or plant or plants or herb or herbs or aromatherap$ or aroma therap$).ti,ab.23. Medicine, Chinese Traditional/ or Plant Preparations/ or Plants, Medicinal/ or Plant Extracts/ or Drugs, Chinese Herbal/24. Aromatherapy/
**5^th^ Concept - study design**25. Health Care Surveys/26. Retrospective Studies/27. Prospective Studies/28. Cohort Studies/29. Observational stud$.ti,ab.30. (prospectiv$ adj3 review$).ti,ab.31. (prospectiv$ adj3 stud$).ti,ab.32. (retrospectiv$ adj3 stud$).ti,ab.33. (retrospectiv$ adj3 review$).ti,ab.34. population-based stud$.ti,ab.35. cohort stud$.ti,ab.36. incidence stud$.ti,ab.37. Sn.fs.38. Ep.fs.39. monitor$.ti,ab.40. surveillance.ti,ab.

The terms within each concept were ORed, and then all 5 concepts were combined using the AND Boolean operator. This search strategy was translated as appropriate for the other databases.

### Selection of studies

#### Screening on title, abstract and full publication stage

Duplicate citations were removed. A study eligibility screening proforma based on pre-specified inclusion criteria was used. Two reviewers (RMDS, EG) independently screened each title and categorised as include, exclude or unsure. The two independent categorisations for all titles were compared and the title categorised again following discussion if two reviewers disagreed. Where there was agreement to exclude, the citation was excluded at this stage. All other citations were reviewed at abstract level. This process was repeated and where there was disagreement, discussion took place between reviewers and citations were re-categorised. Those with agreement to include or as unsure were reviewed at full publication level. The process was repeated at full publication stage. Studies considered as unsure or included at full publication stage were reviewed by a third reviewer (JJK). Reasons for exclusion were documented at the abstract and full paper stage of the screening process.

#### Checking for correct exclusion at each stage

At title stage, two reviewers (RMDS, EG) independently viewed the abstracts for a proportion (2%) of studies excluded. Independent categorisation were compared (as above). This process was repeated at abstract stage where a third reviewer (JJK) reviewed 10% of full papers for studies excluded based on abstract. This was repeated at full publication stage where the same reviewer (JJK) reviewed 20% of excluded full papers. If any studies were excluded incorrectly at any stage, additional checking was performed.

### Data extraction

We extracted the following data from each study:

1. Study characteristics: country; year completed; duration; number of sites; design (prospective or retrospective); clinical setting; number of children.
2. Identification of ADR: definition of ADR, including definition of drug exposure; incidence definition and calculation (numerator and denominator, either at patient or episode level); assessment of causal relationship to drug; person who assessed and categorised ADRs; any method (e.g. case record review) or reporting system used (e.g. Yellow Card).
3. Information relating to the ADR: clinical presentation; associated drug(s)/drug classification; associated risk factors (including age, gender, polypharmacy); ADR considered avoidable.

### Assessment of methodological quality of included studies

As we were unable to find a validated assessment tool for critically appraising observational studies of adverse drug reactions, we developed a quality assessment form specifically for the review. The following aspects were deemed important when assessing study quality: study design; methods for identifying ADRs; methods used to establish the causal relationship between drug and effect; tools for assessing avoidability of the ADR; and tools for assessing severity of the ADR. Criteria were graded as yes, no, unclear, or not reported. Two reviewers (RMDS, EG) independently assessed methodological quality of each study (Table 3).

**Table 3:** Assessment of methodological quality.

**Study design**	
Was the study design clear (prospective, retrospective or combined)?	Yes/No/Unclear/Not reported
**Methods for identifying ADRs**	
Were the methods used to identify ADRs described in sufficient detail?	Yes/No/Unclear/Not reported
Were data collection methods (case-record review, drug chart review, and laboratory data) clearly described?	Yes/No/Unclear/Not reported
Were the individuals (clinicians, self-reported, researchers) who identified ADRs clearly described?	Yes/No/Unclear/Not reported
**Methods for determining causality**	
Was the process of establishing the causal relationship described in detail?	Yes/No/Unclear/Not reported
Were standard methods (validated tool) used in the assessment?	Yes/No/Unclear/Not reported
**Methods for determining avoidability**	
Was the assessment process of establishing avoidability described in detail?	Yes/No/Unclear/Not reported
Were standard methods (validated tool) used in the assessment?	Yes/No/Unclear/Not reported
**Methods for determining severity**	
Was the assessment process of establishing predictability described in detail?	Yes/No/Unclear/Not reported
Were standard methods (validated tool) used in the assessment?	Yes/No/Unclear/Not reported

### Statistical analysis and data synthesis

For each of the three defined populations; children admitted to hospital, children in hospital and children within the community, a forest plot was produced to present the ADR incidence rate and 95% confidence interval for each relevant study. Studies were sub grouped according to whether the incidence rate was reported at the patient and/or episode level and whether or not all patients had been exposed to a drug. Further, for rates reported at the patient level, a distinction was made between studies that had included one admission per patient and those that had included multiple admissions per patient. All results provided per study were included. Pooled estimates were calculated if the variability in incidence rates was not considered too large.

Univariate meta-regression was used to determine if study level characteristics (setting, gender, age, oncology and number of drugs used) are associated with ADR incidence. Incidence rates for ADRs causing admission and occurring in hospital, calculated at the patient level for a single episode were included. Multivariate meta-regression was not undertaken due to the paucity of covariate data. Risk factor analyses reported by any study were collated.

## Results

The search was originally undertaken in November 2009 and retrieved 20 906 potentially relevant citations. An update search was subsequently performed in October 2010 and retrieved an additional 3234 citations. Combining both searches we identified 24 140 potentially relevant citations, of which 5 039 duplicate citations were removed. Screening at title and abstract stage excluded a further 18 592 and 251 citations respectively. Full papers were reviewed and 96 citations met the inclusion criteria. Agreement between reviewers at each stage of the review is described in Figure S1. Additional citations were identified through checking for correct exclusion at each stage (n = 3), reference checking (n = 13) and personal communication with authors (n = 5). In total, 117 citations relating to 102 studies were included in the review (Figure S1).

### Included studies

A total of 102 studies (117 citations), were included in the review. Eighty (80/102) studies described the clinical event as an ADR. In 10 of these studies, ADR was a category within ‘drug related’ problems/admissions; three studies described ADRs as drug induced disease/illness. Sixteen described an ADE where the non-preventable ADE was the same as our definition and two studies used the term iatrogenic disease to describe an ADR. Some studies included multiple settings; 42 studies investigated ADRs as the cause of admission to hospital, 51 studies investigated ADRs in the hospital setting, and 36 studies investigated ADRs in the community setting. Studies included in our review were conducted in 31 different countries, mostly Europe (40/102) and America (32/102). The earliest study assessed the year 1964, the latest assessed years 2008–2009 for causing admission, study size ranged from 24 children to 39,625 admissions. For studies carried out in hospital; the earliest study assessed the year 1964, the latest 2009, study size ranged from 81 children to 64,403 children , and the earliest study assessed the years 1970–1973, the latest 2007, study size ranged from 73 children to 47,107 children for community studies. Characteristics for each individual study are provided in Table 4.

**Table 4:** Study characteristics.

Causing admission studies						
Study	Country	Study duration/design	Clinical setting	Population	Causality assessment	Avoidability assessment
Al-Olah 2008	Saudi Arabia	28 daysProspective	Causing admissionEmergency department	Children and adultsNot reported in publication/unable to obtain from author	Naranjo	Definite preventable and definite non-preventable defined as 3 evaluators in agreement; possible preventable and possible non-preventable 2 in agreement
Classen 1991	USA	18 monthsProspective	Acute care referral hospital	Children and adults0–20 years	Naranjo Score Algorithm	Not reported in publication/unable to obtain from author
Duczmal 2006	Poland	Not reported in publication/unable to obtain from authorRetrospective	Paediatric department	Children0–15 years	Naranjo	Not reported in publication/unable to obtain from author
Easton 1998	Australia	56 daysProspective	Medical ward	Children19 weeks – 18 years	Naranjo Score Algorithm	Schumock and Thornton 1992
Easton-Carter 2004	Australia	22 weeksProspective	Specialist pead teaching hosp and general regional teaching hosp	ChildrenNot reported – 17 years	Dartnell et al 1996	Schumock and Thornton 1992
Gallagher 2010	UK	2 weeksProspective	Large tertiary -paediatric hospital	Children≤18 years	Naranjo	Hallas et al 1990
Gallagher 2011	UK	12 monthProspective	Large tertiary -paediatric hospital	Children≤18 years	NaranjoLiverpool Causality Tool	Hallas et al 1990
Ganeva 2007	Bulgaria	5 yearsProspective	Dermatology and venereology	Children and adults6–18 years	Naranjo Score Algorithm	Not reported in publication/unable to obtain from author
Hewitt 1995	Australia	4 monthsRetrospective	General teaching hospital	Children and adultsAge not reported	Not reported in publication/unable to obtain from author	Not reported in publication/unable to obtain from author
Ives 1987	US	1 yearRetrospective	Family medicine inpatient service at hospital	Children and adults<20 years	Naranjo Score Algorithm	Not reported in publication/unable to obtain from author
Kunac 2009	New Zealand	12 weeksProspective	Paediatric	ChildrenNewborn-16 years	Naranjo Score Algorithm	Schumock and Thornton 1992
Lamabadusuriya 2003	Sri Lanka	11 monthsProspective	Medical ward	ChildrenNot reported in publication/unable to obtain from author	Naranjo Score Algorithm	Not reported in publication/unable to obtain from author
Major 1998	Lebanon	6 monthsProspective	Medical, paediatric	Children and adultsUp to 19 years	Naranjo Score Algorithm	Not reported in publication/unable to obtain from author
McDonnell 2002	US	11 monthsRetrospective	University affiliated teaching hospital	Children and adultsNot reported – 15 years	Naranjo Score Algorithm	Adapted from Schumock &Thornton
Mitchell 1988	US	11 yearsProspective	Teaching and community hospitals	Children0–15 years	Definite - clear implicated drug caused the reaction;Possible – other factors might have caused the reaction.	Not reported in publication/unable to obtain from author
Pouyanne 2000	France	14 daysProspective	Medical, Public hospital	Children and adultsNot reported – 15 years	Not reported in publication/unable to obtain from author	Not reported in publication/unable to obtain from author
Santos 2000	Philippines	3 monthsProspective	Paediatric unit	Children0–18 years	Naranjo Score Algorithm	Not reported in publication/unable to obtain from author
Schneeweiss 2002	Germany	2 yrs and 5 monthsProspective	Internal medicine or emergency departments of all hospitals	Children and adultsAge not provided	Begaud et al 1985	Not reported in publication/unable to obtain from author
Van der Hooft 2006	Netherlands	1 yearRetrospective	Academic and general hospitals	Children and adultsNot reported −<18 years	Not reported in publication/unable to obtain from author	Not reported in publication/unable to obtain from author
Yosselson-Superstine 1982	Israel	7 monthsProspective	General paediatric ward	Children0–16 years	Seidl et al 1965; Seidl et al 1966; Mckenzie 1973; McKenzie 1976; Whyte 1977	Not reported in publication/unable to obtain from author

In hospital studies						
Agarwal et al 2010	USA	4 mthsRetrospective	Paediatric intensive care	Children0–13 years	Not reported in publication/unable to obtain from author	ADEs assessed, non preventable = ADR. Determined by individual sites based on local interpretations, in general was based on the premise that the ADE may have been avoidable, given the appropriate implementation of evidence-based medicine and/or appropriate use of available services
Barstow 1988	US	4 month prospective	Paediatric units	Children and adultsAge not provided	Not reported in publication/unable to obtain from author	Not reported in publication/unable to obtain from author
Benkirane 2009	Morocco	3 month prospective	Intensive care unit	Children and adultsAge not provided	Not reported in publication/unable to obtain from author	ADEs assessed, non preventable = ADR.
Buckley 2007	US	12 daysProspective	Paediatric intensive care	ChildrenNot reported −<18	Not reported in publication/unable to obtain from author	ADEs assessed using Bates et al, non preventable ADE = ADR
Choonara 1984	UK	6 monthsProspective	General paediatric ward	ChildrenNot reported in publication/unable to obtain from author	Seidl et al 1966	6 avoidable: 3 dose prescribed too high, 1 treatment not necessary, 2 application of pharmacological principles would have prevented reactions
Dharnidharka 1993	India	18 monthsProspective	Paediatric unit	Children0–12 years	Stephens et al 1998	Not reported in publication/unable to obtain from author
Dos Santos 2009	Brazil	2 yearsProspective	General paediatric ward	Children1 month–14.4 years	Naranjo	Not reported in publication/unable to obtain from author
dos Santos 2006	Brazil	5 monthsProspective	General paediatric ward	Children1 month–14.4 years	WHO	Not reported in publication/unable to obtain from author
Easton-Carter 2003b	Australia	39 weeksProspective & prospective	General paediatric ward	Children0–17 years	Naranjo Score Algorithm	Schumock and Thornton 1992
Farrokhi 2006	Iran	5 monthsProspective	Paediatric surgery	Children0.5 months–11 years	Not reported in publication/unable to obtain from author	Not reported in publication/unable to obtain from author
Gonzalez-Martin 1998	Chile	1 yearProspective	Paediatric wards	Children5 days–15 years	Naranjo Score Algorithm	Naranjo and Busto 1989
Imbs 1999	France	I dayProspective	Departments of medicine, surgery and geriatrics	Children and adults0–19 years	Two members of the pharmacovigilance team validated each ADR.	Not reported in publication/unable to obtain from author
Jha 2007	Nepal	5 monthsProspective	General paediatric ward	Children and adults0–18 years	Naranjo Score Algorithm	Not reported in publication/unable to obtain from author
Kaushal 2001	US	36 daysProspective	General paediatric ward	Children and adultsNeonates – teenagers	Naranjo Score Algorithm	Not reported in publication/unable to obtain from author
Leach 1998	UK	14 monthsProspective	Regional ICU, a general medical ward, cardiac ICU and cardiac medical ward	ChildrenNot reported in publication/unable to obtain from author	Naranjo, Karch and Lasagna, and Kramer 1979	Not reported in publication/unable to obtain from author
Maistrello 1999	Italy	6 monthsProspective	Emergency ward, Infectivology ward, general paediatric ward , Pneumology ward	Children0–17 years	Not reported in publication/unable to obtain from author	Not reported in publication/unable to obtain from author
Mitchell 1979	US	4 yearsProspective	General medical, oncology, NICU	Children0–17 years	Not reported in publication/unable to obtain from author	Not reported in publication/unable to obtain from author
Neubert 2004	Germany	8 monthsProspective	Paediatric isolation ward	Children5 days–17 years	Naranjo Score Algorithm	Schumock and Thornton 1992
Neubert 2006	Germany	6 monthsProspective	Paediatric isolation ward	Children and adults0–18 years	Naranjo Score Algorithm	Not reported in publication/unable to obtain from author
Shockrollah 2009	Iran	3 monthsProspective	ICU	Children2 days–12 years	Not reported in publication/unable to obtain from author	Not reported in publication/unable to obtain from author
Takata 2008a	USA	3 monthsRetrospective	Paediatric hospitals	Children<18 years	Not reported in publication/unable to obtain from author	Assessed but no detail provided, non preventable ADE = ADR
Takata 2008b	USA	6 monthsProspective	Paediatric teaching hospitals	Children<18 years	Naranjo Score Algorithm	Assessed but no detail provided, non preventable ADE = ADR
Telechea 2010	Uruguay	2 monthsProspective	ICU	Children1 month – 14 years	Karch and Lasagna	Not reported in publication/unable to obtain from author
Turner 1999	UK	13 weeksProspective	Surgical ward, medical ward, neonatal surgical ward, cardiac intensive care unit, general paediatric intensive care units	Children1 day–18 years	Choonara & Harris 1984	Not reported in publication/unable to obtain from author
Uppal 2000	India	3 yearsProspective	General paediatric ward	Children and adultsNot reported in publication/unable to obtain from author	Karch and Lasagna	Not reported in publication/unable to obtain from author
Vazquez de la Villa 1989	Spain	12 monthsProspective	Paediatrics service	Children1–8 years	Naranjo Score Algorithm	Not reported in publication/unable to obtain from author
Wang 2007	US	3 monthsProspective	ICU, General paediatric ward, NICU	ChildrenAge not provided	Not reported in publication/unable to obtain from author	ADEs assessed, non preventable = ADR
Weiss 2002	Germany	8 monthsProspective	Paediatric isolation ward	Children1 month–18 years	adapted Naranjo (Evans et al 1994)	Avoidable or tolerated – toxicity, drug interactions, secondary effects.Unavoidable- idiosyncratic or allergic reactions and intolerance.

Community studies						
Calderon-Ospina 2008	Colombia	12 daysProspective	Accident and Emergency visits	Children and adults0–20 years	WHO	Schumock and Thornton 1992
Campbell 1978	USA	48 monthsProspective	Medical care contacts	Children and adults≤20 years	Not reported in publication/unable to obtain from author	Not reported in publication/unable to obtain from author
Cirko-Begovic 1989	Croatia	3 monthsProspective	General paediatric outpatient unit	Children0–7 years	Hutchinson 1979	Not reported in publication/unable to obtain from author
Dennehy 1996	USA	1 monthRetrospective	Emergency department	Children and adults≤25 years	Strand et al 1990	Considered preventable if avoided through appropriate prescribing, outpatient monitoring or patient compliance.
Doval 1981	India	Not reported in publication/unable to obtain from authorProspective	Outpatient department	Children and adults1 year–20 years	Not reported in publication/unable to obtain from author	Not reported in publication/unable to obtain from author
Easton-Carter 2003a	Australia	18 weeksProspective	Emergency department	Children≤17 years	Dartnell et al 1996	Schumock and Thornton 1992
Horen 2002	France	Not reported in publication/unable to obtain from authorProspective	office-based practice	Children0–15 years	Begaud et al 1985	Not reported in publication/unable to obtain from author
Juntti-Patinen 2006	Finland	6 monthsProspective	Emergency department visits	Children and adultsNot reported in publication/unable to obtain from author	WHO	Not reported in publication/unable to obtain from author
Kaushal 2007	US	2 month blocksProspective	Office based practice	Children<21 years	Not reported in publication/unable to obtain from author	Not reported in publication/unable to obtain from author
Knopf 2010	Germany	3 yearsProspective	Non-clinical community setting	Children≤17 years	WHO	Not reported in publication/unable to obtain from author
Kramer 1985	Canada	1 yearProspective	Private group practice	Children2 days–18.9 years	Kramer 1979	Highly preventable - realistic nondrug alternative available; Probably preventable - safer alternative drug available/lower dosage; Possibly preventable - Dose might have been modified; Unpreventable - would not have changed the choice/dose of drug.
Kushwaha 1994	India	2 yearsProspective	Department of paediatrics	Children0–14 years	Not reported in publication/unable to obtain from author	Not reported in publication/unable to obtain from author
Lemer 2009	USA	10mthsProspective	Attending GP practice	Children≤12 years	ADEs assessed, non preventable = ADR	Not reported in publication/unable to obtain from author
Lewinski 2010	Germany	3 mthsProspective	Community pharmacy	Children and adults≤16 years	Strand et al 1990	Not reported in publication/unable to obtain from author
Martys 1979	UK	2 yearsProspective	General practice	Children and adults2 months–19 years	Not reported in publication/unable to obtain from author	Not reported in publication/unable to obtain from author
Menniti-Ippolito 2000	Italy	1 yearProspective	Family paediatricians	Children0–14 years	Not reported in publication/unable to obtain from author	Not reported in publication/unable to obtain from author
Miller 2006	Australia	10 monthsProspective	General practice	Children and adults≤14 years	Not reported in publication/unable to obtain from author	Thomas & Brennan 2000
Mulroy 1973	UK	1 yearProspective	General practice	Children and adults≤20 years	Not reported in publication/unable to obtain from author	Not reported in publication/unable to obtain from author
Munoz 1998	Spain	25 monthsProspective	Emergency room	Children4 weeks–13 years	Karch and Lasagna	Not reported in publication/unable to obtain from author
Otero Lopez 1999	Spain	6 monthsProspective	Emergency department	Children and adults<15 years	Karch-Lasagna modified algorithm that use the Spanish Pharmacovigilance System.	Schumock and Thornton 1992
Phan 2010	USA	5 mthsRetrospective	Emergency department	Children≤18 years	Naranjo	Not reported in publication/unable to obtain from author
Planchamp 2009	France	6 monthsProspective	Emergency department	Children0–18 years	Begaud et al 1985	Olivier et al 2005
Prince 1992	US	4 monthsRetrospective	Emergency department	Children and adultsAge not provided	Michel and Knodel 1986	Not reported in publication/unable to obtain from author
Rebelo Gomes 2008	Portugal	4 monthsProspective	General paediatric outpatient unit	ChildrenAge not provided	Not reported in publication/unable to obtain from author	Not reported in publication/unable to obtain from author
Sanz 1987	Spain	6 monthsProspective	General practice, outpatient paediatricians	Children<14 years	Karch and Lasagna, Venulet, Dangoumau, Kramer, Naranjo and Blanc	Not reported in publication/unable to obtain from author
Sharma 2007	India	4 monthsProspective	Medicine outpatient department	Children and adults0–20 years	WHO	Not reported in publication/unable to obtain from author
Smith 1997	US	1 monthRetrospective	Emergency department	Children and adults≤18 years	Not reported in publication/unable to obtain from author	Not reported in publication/unable to obtain from author
Stoukides1993	US	6 monthsRetrospective	Emergency department	Children and adults≤20 years	Not reported in publication/unable to obtain from author	Not reported in publication/unable to obtain from author
Valladares 1992	Spain	4 yearsProspective	Ear, nose & throat outpatient unit	Children and adults0–14 years	Karch and Lasagna	Not reported in publication/unable to obtain from author
Woods 1987	UK	26 weeksProspective	Infant care and educational establishments	ChildrenNot reported in publication/unable to obtain from author	Not reported in publication/unable to obtain from author	Not reported in publication/unable to obtain from author
Zahroui 2010	Morocco	7 monthsProspective	Visits to A&E	Children≤16 years	Not reported in publication/unable to obtain from author	Not reported in publication/unable to obtain from author

Combined settings (causing admission & in hospital)						
Baniasadi 2008	Iran	12 month prospective	Multidisciplinary hospital	Children and adults0–18 years	Naranjo Score Algorithm	Schumock and Thornton 1992
Bordet 2001	France	18month prospective	General paediatric ward	Children and adults0–20 years	Begaud et al 1985	Not reported in publication/unable to obtain from author
Fattahi 2005	Iran	5 monthsProspective	Paediatric disease referral centre, paediatric infectious diseases dept	Children0–14 years	WHO	Not reported in publication/unable to obtain from author
Fincham 1989	USA	Not reported in publication/unable to obtain from authorNot reported in publication/unable to obtain from author	Hospital and private practice	Children and adultsAge not provided	Not reported in publication/unable to obtain from author	Not reported in publication/unable to obtain from author
Gill 1995	UK	28 monthsProspective	Paediatric intensive care	Children4 days–16 years	Kramer 1979	Not reported in publication/unable to obtain from author
Haffner 2005	Germany	91 days; 80 days: overlap of 52 daysProspective	ICU, General paediatric ward, Department of Paediatrics	ChildrenAge not provided	WHO	Not reported in publication/unable to obtain from author
Impicciatore 2002	Italy	9 monthsProspective	Paediatric unit	Children3 months–14 years	WHO - confirmed by author	Not reported in publication/unable to obtain from author
Le 2006	US	10 yearsRetrospective	Children's Hospital	Children0–15 years	Definite; Probable; Possible; Conditional	Not reported in publication/unable to obtain from author
Martinez-Mir 1996	Spain	105 days; and 99 daysProspective	Paediatric hospital; Paediatric isolation ward, Lactants B ward	Children1 month–24 months	Spanish Drug Surveillance Scheme (Meyboom 1992)	Not reported in publication/unable to obtain from author
McKenzie 1973	US	8 monthsProspective	University affiliated teaching hospital, paediatric medicine services	Children0 – no upper limit provided	Definite - directly attributable to drugProbable - a known direct relationship Possible - nebulous aspects which could be explained by the illness. No reference provided.	Not reported in publication/unable to obtain from author
McKenzie 1976	US	3 yearsProspective	University affiliated teaching hospital	Children0 – no upper limit provided	Definite - directly attributable to drug.Probable - a known direct relationship. Possible - temporally related to drug. No reference provided.	Not reported in publication/unable to obtain from author
Oshikoya 2007	Nigeria	3 yearsBoth	General paediatric ward	Children4 months–12 years	Jones 1982	Done but no reference provided
Ramesh 2003	India	7 monthsProspective	Memorial hospital	Children and adults0–18 years	WHO	Not reported in publication/unable to obtain from author
Seidl 1966	US	3 monthsProspective	General medical service	Children and adults≤20 years	Documented- confirmatory re-challenge test or a lab result indicating the unwanted effect. Probable - improvement or cessation of symptoms upon withdrawal of drug.	Not reported in publication/unable to obtain from author
Smidt1972	New Zealand	6 monthsProspective	General hospital	Children and adultsNot reported in publication/unable to obtain from author	Not reported in publication/unable to obtain from author	Not reported in publication/unable to obtain from author
Speranza 2008	Uruguay	1 weekProspective	Paediatric hospital	Children0–12 years	Karch and Lasagna	Not reported in publication/unable to obtain from author
Van der Hooft 2008	Netherlands	1 yearRetrospective	Integrated Primary Care Information Database	Children and adultsNot reported-16 years	WHO	Hallas et al 1990
Whyte 1977	UK	10 monthsProspective	Paediatric unit	Children0–12+ (maximum not stated)	Not reported in publication/unable to obtain from author	Not reported in publication/unable to obtain from author

Combined settings (in hospital & in community)						
Doomra 2001	India	15 monthsProspective	General paediatric outpatient unit	Children and adults0–19 years	Naranjo Score Algorithm	Not reported in publication/unable to obtain from author

Combined settings (causing admission, in hospital & community)						
Al-Tajir 2005	United Arab Emirates	12 month prospective	General paediatric ward	Children and adults<15 years	Naranjo Score Algorithm	Schumock and Thornton 1992
Buajordet 2002	Norway	5 month prospective	General paediatric ward	Children0–16 years	Naranjo Score Algorithm	Not reported in publication/unable to obtain from author
Jonville-Bera 2002	France	1 weekProspective	Paediatric wards, A&E, private paediatricians	ChildrenAge not provided	Begaud et al 1985	Not reported in publication/unable to obtain from author
Jose and Padma 2006	India	12 monthsProspective	Various departments (not stated)	Children and adults0–15 years	Naranjo Score Algorithm	Lau et al 2003

### Assessment of methodological quality of included studies

All studies, including those that evaluated ADEs, explicitly stated that they had used either the WHO ADR definition [8] or a comparable one and that they excluded drug errors. Methodological features of each individual study are provided in Table 4.

### Study design

The majority of studies were carried out prospectively (n = 85; 83%), which included 13 in those causing admission, 26 studies with the ADR occurring in hospital, 24 in the community, 16 in hospital and causing admission and 6 in mixed hospital and community settings. Fourteen studies were carried out retrospectively, which included six causing hospital admission, two in hospital studies, and four in the community, one causing admission and in the hospital setting and one the study that considered ADRs that resulted in any medical care contact. Two studies (one in hospital, and one in hospital and causing admission), used both study designs. For the remaining study we were unable to determine the study design (Table 4).

### Persons involved in identifying ADRs

Sixty-four studies reported that a clinician; either a medical doctor, nurse or pharmacist, was involved in the identification of ADRs. Thirty studies reported also involving either the child or parent. Eight studies did not provide information about who identified the ADRs.

### Methods for identifying ADRs

Several methods were used to detect ADRs. Multiple ADR detection methods were employed in 58/102 studies; these consisted of a combination of case record review, drug chart review, laboratory data, computerised ADR reporting system, attendance at ward rounds, and interviewing patients/parents or clinicians. In thirty-one studies case record review alone was undertaken. The remaining eleven studies used; parental interviews/questionnaires (5 studies), clinical assessments (3 studies), clinician questionnaires (1 study), ward round (1 study) and a nationwide computer database (1 study). The remaining study report did not refer to the methods used.

### Studies estimating the proportion of paediatric hospital admissions related to ADRs

#### Description of studies

There were 42 studies, where ADRs have been investigated as the cause of admission to hospital. The period under study varied widely and ranged from 1 week to 11 years. The majority of studies were described as being performed in a general paediatric unit or ward (n = 22) [9]–[29], [34]. Four studies included general medicine [30]–[33] one study in a hospital emergency department [35]. Two studies covered general medicine and a hospital emergency department, [36], [37], and one study an integrated primary care information database [38]. Two studies were performed in the paediatric intensive care setting [39], one in combination with general paediatrics also [40]. Seven studies covered a combination of clinical settings [41]–[47]. The remaining three studies were performed in dermatology and venereology [48], Infectious diseases [49] and an isolation ward [50].

#### ADR incidence

**Figure 1:**
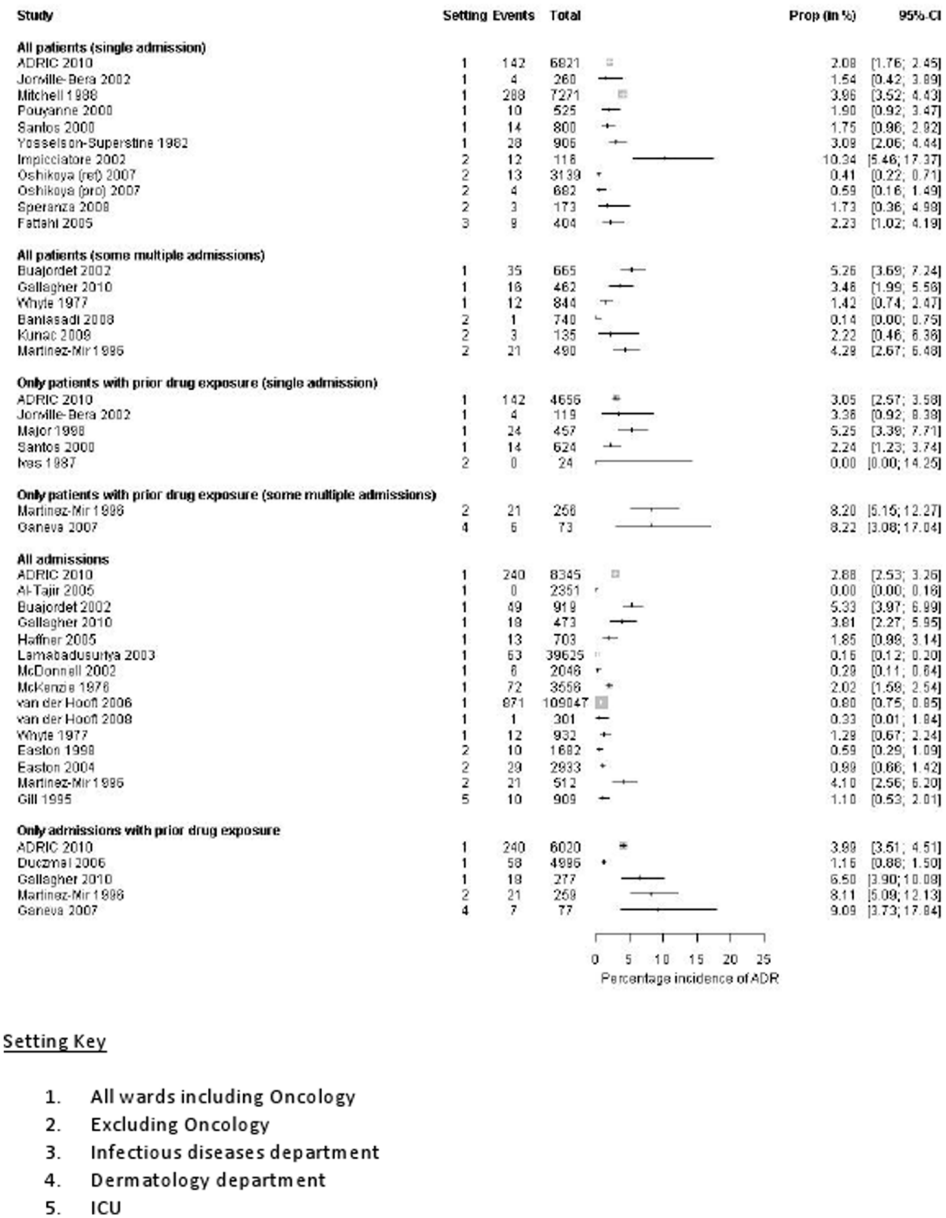
What proportion of all paediatric hospital admissions are ADR related?

We do not have ADR incidence rates for 12/42 of these studies as the child only data was not available (n = 4), data were not split by clinical setting (n = 5), data provided for ADRs in hospital but not causing admission (n = 2) and data were provided for the total number of ADRs but not the ADR frequency at the patient or episode level (n = 1). Figure 1 presents data from all studies that provide incidence rates for ADRs causing admission to hospital (n = 30). These rates range from 0.4% to 10.3% of children (single admission). One study was an extreme outlier [20] and if this was excluded we found a reduction in the upper limit of this range to 4%, and a pooled incidence estimate of 2.9% (2.6%, 3.1%).

### Studies estimating the proportion of children experiencing an ADR during their admission

#### Description of studies

We have included 51 studies, where ADRs have been investigated in the hospital setting. The period under study varied widely and ranged from 1 day to ten years. The majority of studies where described as being performed in a general paediatric unit or ward (n = 24) [14], [19], [20], [22]–[26], [28], [34], [37], [51]–[54], [56]–[63], [85] two of which included intensive care also [64], [40]. Six studies were performed solely in the intensive care setting [39], [65]–[69], one of which included general medicine [70]. Three studies included children on an isolation ward [71]–[73]. One study was performed using an integrated primary care information database [38] and one in an isolation ward [50]. The remaining thirteen studies covered a combination of clinical settings [41], [43]–[47], [49], [74]–[79].

#### ADR incidence

**Figure 2:**
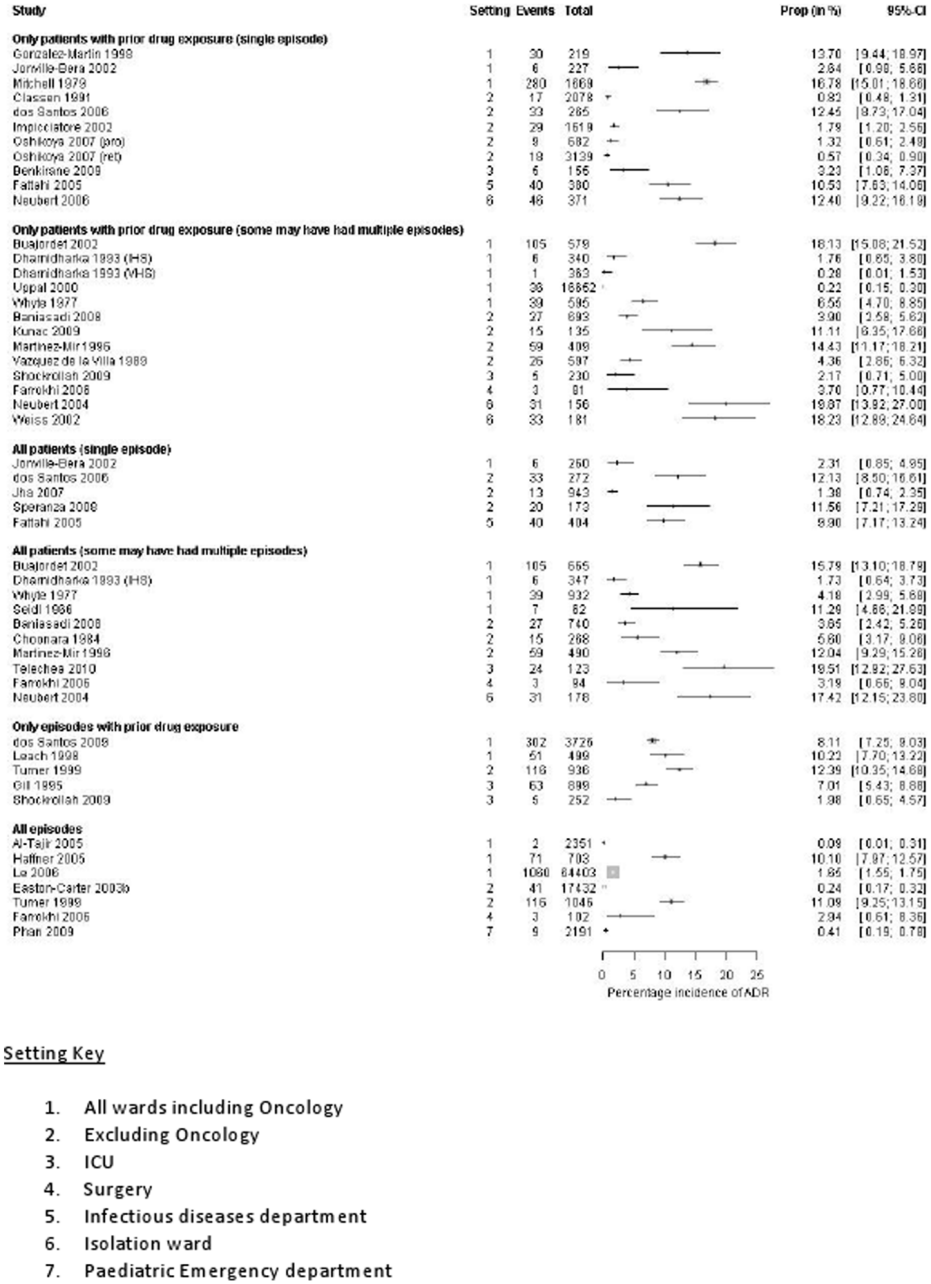
What proportion of children in hospital experience an ADR during their admission?

We do not have ADR incidence rates for 18/54 of these studies as the child only data was not available (n = 3), the data were not split by clinical setting (n = 7), data were provided for the total number of ADRs but not the ADR frequency at the patient or episode level (n = 5), data provided for ADRs and ADEs combined (n = 2), and data provided for ADRs causing admission but not in hospital (n = 1). Figure 2 presents data from all studies that provide incidence rates for ADRs in hospital (n = 36). These estimates range from 0.6% to 16.8% of patients (at a single episode and with prior drug exposure). A pooled estimate has not been calculated since the rates are considered too varied.

### Studies estimating the incidence of ADRS in outpatient children

#### Description of studies

We have included 36 studies, where ADRs have been investigated in the community setting. The period under study varied widely and ranged from 1 week to 11 years. The majority of studies where described as being performed in a hospital outpatient or accident emergency department (n = 21) [25], [25], [47], [55], [78], [80]–[84], [86]–[97]. Nine studies were performed in general practice [98]–[106]. The remaining six studies were performed in an infant care and educational establishment [107], local community setting [108], [109], general practice and accident and emergency department [37], outpatient population seeking medical care [110], and after discharge from hospital [26].

#### ADR incidence

**Figure 3:**
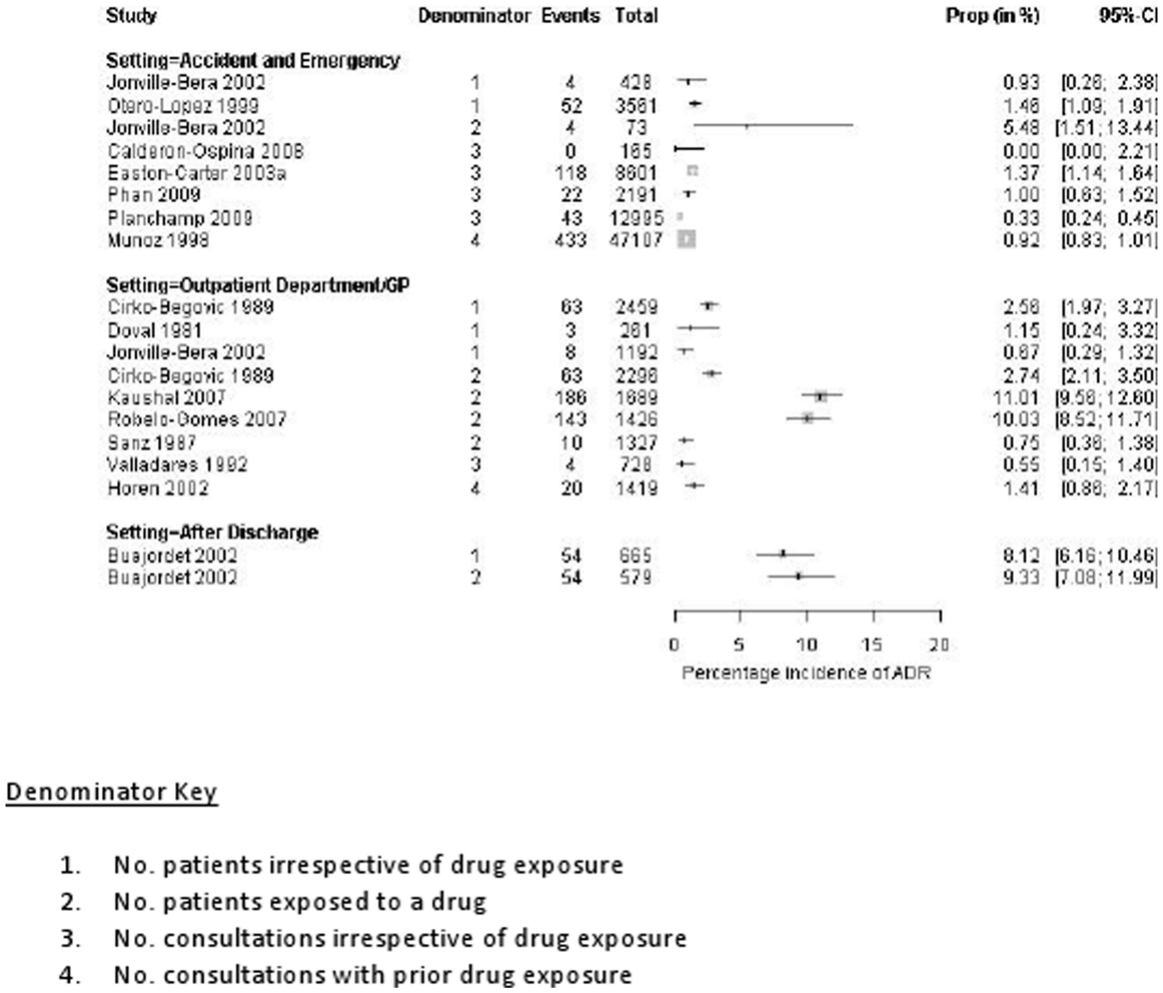
What proportion of outpatient children experience ADRs?

We do not have ADR incidence rates for 19 (19/36) of these studies as the child only data were not available (n = 10), the data were not split by clinical setting (n = 3), data not available for the total number of children/visits (n = 4), data were provided for the total number of ADRs but not the ADR frequency at the patient or visit level (n = 1) and data were provided for errors only (n = 1). Figure 3 presents data from studies that provide incidence rates for ADRs in the community (n = 15). Two studies were not included in this figure due to their method of ADR ascertainment,

### All Settings

#### Drugs and clinical presentation associated with ADR

We do not have information on the drugs involved in ADRs for 50/102 studies, as the child only data were not available (37 studies), ADRs were a subset of events looked at and ADR specific data were not reported (10 studies), and drug data were not available in the publication (3 studies). For studies that provided data (52/101) (Table 5); anti-infectives were the drug class most commonly reported across the three settings. Proportions ranged from 3.5%–66.6% for causing admission studies (17 studies); 8.6%–100% for in hospital studies (24 studies); and 17%–78% for community studies (13 studies). The most common associated clinical presentations reported were nausea, vomiting, diarrhoea and skin rash. Anti-epileptics were the second most common reported drug class in both the causing admission and in hospital studies; proportions ranging from 0.8%–30% (12 studies); and 3.9%–46.6% (14 studies) respectively. Reported clinical presentations were ataxia, skin rash, increased fitting, and drowsiness. Non-steroidal anti-inflammatory drugs (NSAIDs) were frequently reported as being associated with ADRs in studies in children in both the causing admission and outpatient studies, proportions ranging from 4.1%–25% (9 studies) and 1%–10% (6 studies) respectively. Reported clinical presentations were cutaneous reactions, haematuria, hypertranspiration, drowsiness, abdominal pain, aggressiveness and vomiting.

**Table 5:** Drug class and clinical presentation of ADRs.

Causing admission studies					
Drug class	Study	Population of study	Total number of ADRs reported in study	Number of ADRs due to drug class (%)	Clinical presentation
**Anti-infectives** **(n- = 16)**					
	Easton (1998)	1682 admissions	10	1 (10%)	Colitis, ileus
	Impicciatore (2002)	116 children	12	4 (33.3%)	Urticaria, periorbital oedema, neutropenia
	Lamababusuriya (2003)	39625 admissions	63	38 (60.3%)	Erythema multiforme, stevens-johnson syndrome, rash, raised intracranial pressure
	Oshikoya (2007)	3821 children	17	7 (41.1%)	Provided for deaths only ×1
	Easton Carter (2004)	2933 admissions	29	Not reported in publication	Not reported in publication
	Mitchell (1988)	7271 children	288	10 (3.5%)	Diarrhoea, fever, erythema multiforme death ×2
	Major (1998)	457 children	26	6 (23%)	Not reported in publication
	Santos (2000)	624 children	14	6 (42.8%)	Not reported in publication
	Gallagher (2010)	462 children	18	3 (16.6%)	Diarrhoea
	Duczmal (2006)	4996 admissions	58	Not reported in publication	Not reported in publication
	Ganeva (2007)	73 children	6	4 (66.6%)	Not reported in publication
	Fattahi (2005)	404 children	9	4 (44.4%)	Not reported in publication
	Martinez-Mir (1996)	490 children	21	10 (47.6%)	Not reported in publication
	Yosselson-Superstine (1982)	906 children	29	Not reported in publication	Not reported in publication
	McKenzie (1976)	3556 admissions	72	Not reported in publication	Provided for deaths only ×2
	Gallagher (2011)	6821 children	249	16 (6.4%)	Diarrhoea, Rash, Vomiting, Lip swelling, Deranged LFTs, Thrush
**Anti-epileptics** **(n = 12)**					
	Easton (1998)	1682 admissions	10	3 (30%)	Increased fitting, Rash, aphasia/motor regression
	Impicciatore (2002)	116 children	12	2 (16.6%)	coma
	Lamababusuriya (2003)	39625 admissions	63	4 (6.3%)	Ataxia and cerebellar signs, liver failure, stevens-johnson syndrome
	Oshikoya (2007)	3821 children	17	1 (5.8%)	Not reported in publication
	Mitchell (1988)	7271 children	288	23 (7.9%)	Lethargy, ataxia, rash, erythema
**Anti-epileptics**					
	Le (2006)	64 403 admissions	35	Not reported in publication	Not reported in publication
	Santos (2000)	624 children	14	1 (7.1%)	Not reported in publication
	Yosselson-Superstine (1982)	906 children	29	Not reported in publication	Not reported in publication
	McKenzie (1976)	3556 admissions	72	Not reported in publication	Not reported in publication
	Fattahi (2005)	404 children	9	1 (11.1%)	Not reported in publication
	Jonville-Bera (2002)	260 children	4	1 (25%)	Convulsion
	Gallagher (2011)	6821 children	249	2 (0.8%)	Constipation, respiratory depression
**NSAIDS** **(n = 9)**					
	Duczmal (2006)	4996 admissions	58	Not reported in publication	Not reported in publication
	Impicciatore (2002)	116 children	12	1 (8.3%)	Coma
	Lamababusuriya (2003)	39625 admissions	63	3 (4.7%)	Rectal bleeding, Aspirin – Reye syndrome
	Major (1998)	457 children	26	2 (7.6%)	Not reported in publication
	Gill (1995)	909 admissions	10	1 (10%)	Not reported in publication
	Gallagher (2011)	6821 children	249	31 (12.4%)	Post-op bleeding, haematemesis, constipation, abdominal pain
	Gallagher (2010)	462 children	18	1 (5.5%)	Haematemesis
	Mitchell (1988)	7271 children	288	12 (4.1%)	Gastritis
	Jonville-Bera (2002)	260 children	4	1 (25%)	Melaena
**Cytotoxics** **(n = 8)**					
	Mitchell (1988)	7271 children	288	Not reported in publication	Deaths ×2
	Major (1998)	457 children	26	10 (38.4%)	Not reported in publication
	Santos (2000)	624 children	14	2 (14.2%)	Not reported in publication
	Yosselson-Superstine (1982)	906 children	29	Not reported in publication	Death ×1
	McKenzie (1976)	3556 admissions	72	Not reported in publication	Provided for deaths only ×3
	Fattahi (2005)	404 children	9	2 (22.2%)	Not reported in publication
	Gallagher (2010)	6821 children	249	110 (44.2%)	Thrombocytopenia, Anaemia, Vomiting, Mucositis, Deranged LFTs, Immunosuppression, Diarrhoea, Nausea, Constipation, Headache, Abdominal pain, Back pain, Haematuria, Leukencephalopathy, Deranged renal function
	Gallagher (2010)	462 children	18	9 (50%)	Pyrexia, neutropenia, lethargy, decreased responsiveness, vomiting
**Corticosteroids** **(n = 7)**					
	Easton (1998)	1682 admissions	10	1 (10%)	Unstable diabetes
	Santos (2000)	624 children	14	1 (7.1%)	Upper GI bleed
	Yosselson-Superstine (1982)	906 children	29	Not reported in publication	Not reported in publication
	McKenzie (1976)	3556 admissions	72	Not reported in publication	Not reported in publication
	Ganeva (2007)	73 children	6	2 (33.3%)	Not reported in publication
	Gallagher (2010)	6821 children	249	102 (41.0%)	Immunosuppression, Post-op bleeding, Hyperglycaemia, Hypertension, Gastritis, Increased appetite, Impaired healing, adrenal suppression
	Gallagher (2010)	462 children	18	1 (5.5%)	Vomiting
**Vaccines** **(n = 7)**					
	Easton (1998)	1682 admissions	10	1 (10%)	Hypotonic-hyporesponsive episode
	Lamababusuriya (2003)	39625 admissions	63	9 (14.2%)	Rash, encephalopathy, fits, head lag
	Easton Carter (2004)	2933 admissions	29	Not reported in publication	Not reported in publication
	Mitchell (1988)	7271 children	288	5 (1.7%)	Not reported in publication
	Santos (2000)	624 children	14	1 (7.1%)	Not reported in publication
	Gill (1995)	909 admissions	10	2 (20%)	Seizures, fever
	Gallagher (2010)	6821 children	142		Fever, Rash, Irritability , Seizure , Vomiting, Pallor, Apnoea , Limb swelling, Lethargy , Thrombocytopenia Diarrhoea, Abdominal pain, Respiratory distress, Kawasaki disease

In hospital studies					
**Anti-infectives** **(n = 24)**					
	Al-Tajir (2005)	2351 episodes	2	2 (100%)	Not reported in publication
	Baniasadi (2008)	693 children	27	Not reported in publication	Not reported in publication
	Choonara (1984)	268 children	15	5 (33.3%)	Vomiting, oral monilia, diarrhoea
	Dharnidharka (1993)	703 children	7	1 (14.2%)	Skin rash
	Dos Santos (2006)	265 children	47	18 (38.2%)	Not reported in publication
	Dos Santos (2009)	3726 episodes	302	57 (18.8%)	Not reported in publication
	Easton Carter (2003b)	17432 episodes	41	Not reported in publication	Not reported in publication
	Farrokhi (2006)	81 children	3	1(33.3%)	Not reported in publication
	Fattahi (2005)	380 children	40	35 (87.5%)	Not reported in publication
	Gill (1995)	899 episodes	76	15 (19.7%)	Not reported in publication
	Gonzalez-Martin (1998)	219 children	46	4 (8.6%)	Not reported in publication
	Jha (2007)	943 children	13	12 (92.3%)	Macupapular rashes, vomiting, diarrhoea, drug fever
	Jonville-Bera (2002)	227 children	6	2 (33.3%)	Diarrhoea, rash
	Impicciatore (2002)	1619 children	29	9 (31.0%)	Urticaria, increased transaminase levels, vomiting, diarrhoea,
	Le et al (2006)	64 403 admissions	1060	Not reported in publication	Not reported in publication
	Leach (1998)	499 episodes	58	23 (39.6%)	Vomiting, rash, diarrhoea, arthropathy, neutropenia, nausea, fits
	Mitchell (1979)	1669 children	280	Not reported in publication	Not reported in publication
	Maistrello (1999)	1103 children	59	24 (40.6%)	Gasto-intestinal disorders,
	Martinez-Mir (1996)	490 children	68	Not reported in publication	Not reported in publication
	Neubert (2004)	156 children	31	Not reported in publication	Not reported in publication
	Oshikoya (2007)	3821 children	27	12 (44.4%)	Red man syndrome, pustular rash, stevens-johnson syndrome, erythema, jaundice, anaphylaxis, urticaria, fever
	Shockrollah (2009)	230 children	5	2 (40%)	Not reported in publication
	Turner (1999)	936 episodes	157	34 (21.6%)	Not reported in publication
	Vazquez de la villa (1999)	597 children	26	9 (34.6%)	Diarrhoea, vomiting, rash
**Anti-epileptics** **(n = 14)**					
	Choonara (1984)	268 children	15	7 (46.6%)	Drowsiness, hyperactivity, ataxia
	Dharnidharka (1993)	703 children	7	1 (14.2%)	Skin rash
	Dos Santos (2009)	3726 episodes	302	26 (8.6%)	Not reported in publication
	Easton Carter (2003b)	17432 episodes	41	Not reported in publication	Not reported in publication
	Gill (1995)	899 episodes	76	3 (3.9%)	Not reported in publication
	Gonzalez-Martin (1998)	219 children	46	5 (10.8%)	Not reported in publication
	Le et al (2006)	64 403 admissions	1060	Not reported in publication	Not reported in publication
	Leach (1998)	499 episodes	58	1	Apnoea
	Mitchell (1979)	1669 children	280	Not reported in publication	Not reported in publication
	Martinez-Mir (1996)	490 children	68	Not reported in publication	Not reported in publication
	Neubert (2004)	156 children	31	Not reported in publication	Not reported in publication
	Oshikoya (2007)	3821 children	27	2 (7.4%)	Erythema
**Anti-epileptics**					
	Telechea (2010)	123 children	46	15 (32.6%)	Not reported in publication
	Vazquez de la villa (1999)	597 children	26	4 (15.3%)	Sedation, paradoxil reaction
**Corticosteroids** **(n = 10)**					
	Dos Santos (2006)	265 children	47	11 (23.4%)	Not reported in publication
	Gill (1995)	899 episodes	76	6 (7.8%)	Not reported in publication
	Gonzalez-Martin (1998)	219 children	46	3 (6.5%)	Not reported in publication
	Impicciatore (2002)	1619 children	29	1 (3.4%)	Rash
	Leach (1998)	499 episodes	58	1 (1.7%)	Gastric irritation
	Mitchell (1979)	1669 children	280	Not reported in publication	Not reported in publication
	Neubert (2004)	156 children	31	Not reported in publication	Not reported in publication
	Telechea (2010)	123 children	46	4 (8.6%)	Not reported in publication
	Turner (1999)	936 episodes	157	10 (6.3%)	Not reported in publication
	Vazquez de la villa (1999)	597 children	26	1 (3.8%)	Cushing syndrome
**Bronchodilators** **(n = 9)**					
	Choonara (1984)	268 children	15	3 (20%)	Tachycardia
	Easton Carter (2003b)	17432 episodes	41	Not reported in publication	Not reported in publication
	Gill (1995)	899 episodes	76	8 (10.5%)	Not reported in publication
	Gonzalez-Martin (1998)	219 children	46	8 (17.3%)	Not reported in publication
	Impicciatore (2002)	1619 children	29	5 (17.2%)	Tremor, tachycardia
	Neubert (2004)	156 children	31	Not reported in publication	Not reported in publication
	Telechea (2010)	123 children	46	8 (17.3%)	Not reported in publication
	Turner (1999)	936 episodes	157	8 (5.0%)	Not reported in publication
	Vazquez de la villa (1999)	597 children	26	11 (42.3%)	Tachycardia, nervousness, vomiting
**Cytotoxics** **(n = 7**					
	Dos Santos (2009)	3726 episodes	302	10 (3.3%)	Not reported in publication
	Gonzalez-Martin (1998)	219 children	46	7 (15.2%)	Not reported in publication
	Jonville-Bera (2002)	227 children	6	4 (66.6%)	Vomiting
**Cytotoxics**					
	Le et al (2006)	64 403 admissions	1060	Not reported in publication	Not reported in publication
	Leach (1998)	499 episodes	58	1 (1.7%)	Thrombocytopenia
	Mitchell (1979)	1669 children	280	Not reported in publication	Not reported in publication
	Telechea (2010)	123 children	46	1 (2.1%)	Not reported in publication
**Diuretics** **(n = 6)**					
	Easton Carter (2003b)	17432 episodes	41	Not reported in publication	Not reported in publication
	Leach (1998)	499 episodes	58	1 (1.7%)	Over diereses
	Mitchell (1979)	1669 children	280	Not reported in publication	Not reported in publication
	Neubert (2004)	156 children	31	Not reported in publication	Not reported in publication
	Telechea (2010)	123 children	46	9 (19.5%)	Not reported in publication
	Turner (1999)	936 episodes	157	31 (19.7%)	Not reported in publication

Community					
**Anti-infectives** **(n = 13)**					
	Cirko-Begovic (1989)	2459 children	63	49 (78%)	Not reported in publication
	Easton-Carter (2003a)	8601 consultations	118	Not reported in publication	Not reported in publication
	Horen (2002)	1419 consultations	20	9 (45%)	Not reported in publication
	Juntti-Patinen (2006)	Not reported for children	4	Not reported for children only	Not reported for children only
	Kaushal (2007)	1689 children	226	158 (70%)	Nausea, vomiting and diarrhoea.
	Kramer (1985)	4244 courses of therapy	200	Not reported in publication	Diarrhoea, other gastrointestinal complaints and skin rashes
	Menniti-Ippolito (2000)	7890 children	119	79 (66%)	Cutaneous, gastrointestinal, eosinophilia, neurological, angioedema, fever
	Planchamp (2009)	12995 consultations	43	Not reported in publication	Not reported in publication
	Sanz (1987)	1327 children	10	4 (40%)	Cutaneous reaction and diarrhoea
	Munoz (1998)	47107 consultations	447	49.5%	Included skin reactions
	Jonville-Bera (2002)	A&E: 428 childrenPrivate paediatricians: 1192 children	A&E: 4Private paediatricians: 8	A&E: 2 (50%)Private paediatricians: 6 (75%)	Diarrhoea, rash, vomiting
**Anti-infectives**					
	Woods (1987)	1590 children	235	40 (17%)	Diarrhoea, drowsiness, rash, headache, hyperactivity, anorexia, abdominal pain, vomiting, sleep disturbance
	Zahraoui (2010)	Not reported	24	Not reported in publication	Not reported in publication
**NSAIDs** **(n = 6)**					
	Kaushal (2007)	1689 children	226	2 (1%)	Not reported in publication
	Menniti-Ippolito (2000)	7890 children	119	3 (3%)	Cutaneous, haematuria, hypertranspiration
	Munoz (1998)	47107 consultations	447	Not reported	Not reported in publication
	Planchamp (2009)	12995 consultations	43	Not reported in publication	Not reported in publication
	Sanz (1987)	1327 children	10	1 (10%)	Not reported in publication
	Woods (1987)	1590 children	235	9 (4%)	Drowsiness, abdominal pain, aggressiveness, vomiting
**Analgesics** **(n = 5)**					
	Kaushal (2007)	1689 children	226	1 (0.4%)	Not reported in publication
	Munoz (1998)	47107 consultations	447	Not reported in publication	Not reported in publication
	Planchamp (2009)	12995 consultations	43	Not reported in publication	Not reported in publication
	Woods (1987)	1590 children	235	11 (5%)	Drowsiness, irritability, aggressiveness
	Zahraoui (2010)	Not reported	24	Not reported in publication	Not reported in publication
**Vaccines** **(n = 5)**					
	Horen (2002)	1419 consultations	20	5 (25%)	Not reported in publication
	Jonville-Bera (2002)	A&E: 428 childrenPrivate:1192 children	A&E:4Private:8	A&E: 1 (25%)Private: 2 (25%)	A&E: rashPrivate:fever
	Menniti-Ippolito (2000)	7890 children	119	14 (12%)	Not reported in publication
	Munoz (1998)	47107 consultations	447	? 9.2%	Not reported in publication
	Planchamp (2009)	12995 consultations	43	Not reported in publication	Not reported in publication
**Antihistamine** **(n = 4)**					
	Cirko-Begovic (1989)	2459 children	63	2 (3%)	Not reported in publication
	Kaushal (2007)	1689 children	226	2 (1%)	Not reported in publication
	Menniti-Ippolito (2000)	7890 children	119	2 (2%)	Not reported in publication
	Woods (1987)	1590 children	235	46 (20%)	Drowsiness, aggressiveness, dry mouth, headache, irritability, diarrhoea
**Bronchodilators** **(n = 3)**					
	Kaushal (2007)	1689 children	226	16 (7%)	Not reported in publication
	Kramer (1985)	4244 courses of therapy	200	Not reported in publication	Various manifestations of central nervous stimulation
	Woods (1987)	1590 children	235 ADRs	6 (3%)	Hyperactivity, shakiness, dizziness, irritability, sleep disturbance.
**Steroid** **(n = 3)**					
	Horen (2002)	1419 consultations	20	1 (0.05%)	Not reported in publication
	Kaushal (2007)	1689 children	226	12 (5%)	Not reported in publication
	Woods (1987)	1590 children	235	5 (2%)	Abdominal pain, diarrhoea
					

Combined settings (causing admission & in hospital)					
**Anti-infectives** **(n = 2)**					
	Haffner (2006)	703 admissions	101	Not reported in publication	Not reported in publication
	Speranza (2008)	173 children	24	10 (41.6%)	Not reported in publication
**Bronchodilators** **(n = 1)**					
	Haffner (2006)	703 admissions	101	Not reported in publication	Not reported in publication
**Anti-epileptics** **(n = 2)**					
	Haffner (2006)	703 admissions	101	Not reported in publication	Not reported in publication
	Speranza (2008)	173 children	24	4 (16.6%)	Not reported in publication
**Cardiovascular** **(n = 1)**					
	Haffner (2006)	703 admissions	101	Not reported in publication	Not reported in publication
**Analgesics** **(n = 1)**					
	Speranza (2008)	173 children	24	2 (8.3%)	Not reported in publication
**Anti-ulcer** **(n = 1)**					
	Speranza (2008)	173 children	24	2 (8.3%)	Not reported in publication
**Psychotropic** **(n = 1)**					
	Speranza (2008)	173 children	24	2 (8.3%)	Not reported in publication

Combined settings (in hospital & community)					
**Anti-infectives** **(n = 1)**					
	Kushwaha (1994)	20310 admissions	267	Not reported in publication	Erythmatous maculopapular rash, thrombophlebitis, erythema multiformae, fixed drug reaction, urticaria, jaundice, aplastic anaemia, thrombocytopaenia purpura
**Vaccines** **(n = 1)**					
	Kushwaha (1994)	20310 admissions	267	Not reported in publication	Nodular cyst in gluteal region, injection abcess
**NSAID** **(n = 1)**					
	Kushwaha (1994)	20310 admissions	267	Not reported in publication	Erythmatous maculopapular rash
**Analgesic** **(n = 1)**					
	Kushwaha (1994)	20310 admissions	267	Not reported in publication	Erythmatous maculopapular rash, urticaria
**Steroid** **(n = 1)**					
	Kushwaha (1994)	20310 admissions	267	Not reported in publication	injection abscess

Combined settings (causing admission, in hospital & community)					
**Steroid** **(n = 1)**					
	McKenzie (1973)	658 children	175	Not reported in publication	Psychotic reaction, cushingoid syndrome, cataracts, hypertension
**Anti-infectives** **(n = 1)**					
	McKenzie (1973)	658 children	175	Not reported in publication	Rash, diarrhoea, facial flush, monilia, pain in injection site
**Cytotoxics** **(n = 1)**					
	McKenzie (1973)	658 children	175	Not reported in publication	Alopecia, peripheral neutitis, mouth ulcer, injection site inflammation, leukopenia, secondary infection

Note 1 patient in the Zahraoui (2010) study died (gastrointestinal bleeding and severe thrombocytopenia after prolonged anti-convulsant treatment.

Mitchell (1988) – 5 deaths (fever, vomiting, arrhythmia and cardiopulmonary arrest attributed to theophylline and erythromycin; cardiac arrest and hypernatremia attributed to halothane and nitrous oxide pneumonia attributed to chemotherapy-induced immunosuppression; cardiotoxicity attributed to doxorubicin; candida sepsis and meningitis attributed to chemotherapy-induced immunosuppression).

Yosselson-Superstine (1982) – 1 death (no detail provided).

In addition, corticosteroids were commonly reported across the three settings. Proportions ranging from 5.5%–41.0% for causing admission studies (7 studies); 1.7%–23.4% for in hospital studies (10 studies); and 0.05%–5% for community studies (3 studies). The most common associated clinical presentations reported were immunosuppression, post-operative bleeding, gastric irritation, and diarrhoea.

The distribution of drugs implicated in ADRs reflect the prescribing practices for the individual settings. For example; vaccines were commonly reported in causing admission studies (7 studies) and community studies (5 studies). Proportions ranged from 1.7%–20.0% and 9.2%–25% respectively, with rash and fever being the most common associated clinical presentations. Cytotoxics were reported in both causing admission (8 studies) and in hospital studies (7 studies), proportions ranged from 14.2%–50%, and 1.7%–66.6% respectively. The remaining studies reported a variety of drugs implicated in ADRs, for some more than one drug was the cause of a single ADR (Table 5).

### Meta-regression

Univariate meta-regression results (Table 6) suggest that the incidence rate for ADRs occurring in hospital is higher than for ADRs causing admission (OR = 2.73 (0.93, 8.03)). In addition, the results suggest that the incidence rate is higher for studies with a relatively high mean/median number of drugs per patient (OR = 1.49 (1.14, 1.94)), high percentage of females (OR = 1.13 (0.91, 1.40)), high percentage of oncology patients (OR = 1.15 (0.89, 1.50)) and low mean age of patients (OR = 0.71 (0.39, 1.27)). However, only the variable representing the mean/median number of drugs per patient achieves statistical significance.

**Table 6:** Univariate meta-regression results for causing admission and in hospital incidence rates.

Covariate	OR (95% CI)	P
Setting: Admission	1	
Hospital	2.73 (0.93,8.03)	0.07
% Female patients	1.13 (0.91,1.40)	0.23
Mean age (years)	0.71 (0.39,1.27)	0.21
Mean/median number of drugs	1.49 (1.14,1.94)	0.01
% Oncology patients	1.15 (0.89,1.50)	0.25

### Risk factors

Risk factor analyses reported by all studies were collated. Consistent with the meta-regression results, evidence is provided, from 10/19 studies that consider gender as a risk factor, that boys are less likely to have an ADR, and, from 16/17 studies, that risk increases with the number of drugs taken. In addition, 3/3 studies suggest that the risk of ADRs is greater with off-label use. Only two studies considered oncology as a risk factor. The results for the age analyses do not follow a clear pattern and are difficult to interpret due to the variety of age categorisations used.

### Tools for assessing causality

Nearly three quarters of the studies (72/102) mentioned a causality assessment, of which the Naranjo algorithm was the most frequently used tool (30/72). Of the 72 studies, seven used a self-assessment method rather than a published causality tool. Despite the majority of studies mentioning a causality assessment, only half of these studies (36/72) reported causality data that were complete for all identified ADRs, specific to the paediatric population and did not include errors as part of the assessment (Table 4).

### Tools for assessing severity

Thirty-four (34/102) studies performed an ADR severity assessment. Rates ranged from 0%–66.7% of reported ADRs considered to be severe. By setting, the proportion of ADRs occurring in hospital assessed as severe ranged from 0% to 66.7%, compared with 0% to 45.5% of ADRs causing admission, and 0% to 32.6% of ADRs occurring in the community. Twenty studies provided a reference to indicate the severity tools used, however tools differed widely. Examples of ADRs assessed as severe were those that caused death or were directly life-threatening, caused hospital admission, prolonged hospitalisation or caused transfer to higher level of clinical care.

### Assessment of avoidability

Nineteen (19/101) studies performed an avoidability assessment, however, data were only available for 14/19 studies as child only data were not available in 4/19 and ADR specific data were not provided in 1/19. For these 14 studies 7%–98% of ADRs were designated either definitely/possibly avoidable. Three studies provided the rationale for sixty-two avoidable ADRS; inappropriate selection or indication for use of drug (n = 14), inadequate patient education (n = 14), prescribing not rationale (n = 11), lack of appropriate prophylaxis for known ADR (n = 9), lack of appropriate monitoring of drugs (n = 5), previous known ADR to medication (n = 3), dose prescribed was too high (n = 3), inappropriate duration of treatment (n = 1), drug was not prescribed per treatment protocol (n = 1), inappropriate duration of drug and monitoring of treatment (n = 1). Ten studies used a recognised avoidability assessment; of which half used Schumock and Thornton [111] (Table 4).

## Discussion

This is the largest systematic review of ADRs in children to date and shows clearly that ADRs are an important clinical problem for children and have been the subject of a large number of studies.

Unlike previous systematic reviews [3], [4], [5], our review searched for studies using a comprehensive search strategy of a large number of databases, including those specific to toxicology and pharmacology. Nineteen databases were searched of which eight retrieved eligible studies. When compared with the previous reviews this resulted in an additional 73 studies being included in our review, of which, in 24, we were able to extract data. We included studies where ADEs had been evaluated, and that included both adults and children. In addition, we contacted authors of studies to obtain unpublished information. As a result, we were able to obtain unreported ADR incidence data for an additional 24/102 studies. This allowed us to make a more informed judgement regarding ADR incidence estimates.

In agreement with previous studies, including those specific to adults [112], this review found that ADR incidence rates were generally higher in hospitalised children than ADR rates causing hospital admission or in an outpatient setting. One of the main difficulties when comparing ADR incidence rates, particularly from observational studies, is that the studies differ in a number of ways, such as clinical setting, population characteristics and study duration. This may explain the large variation in the incidence rates reported. However, since the numerators and denominators used to calculate ADR incidence were not consistent across studies it was not possible to apply statistical methods to comprehensively explore the heterogeneity. Due to the large amount of heterogeneity, a pooled estimate of the incidence rate has been provided for ADRs causing admission only.

Concerning risk factors associated with ADRs, we found evidence, from both univariate meta-regression and the collation of risk factor analyses from individual studies, that the use of multiple drugs is an important predictor of ADRs. This may be due to the additive risk of an ADR when receiving several drugs or to drug-drug interactions.

We report where possible the drugs associated with ADRs and the clinical presentation, although information regarding drugs involved was poorly reported. The types of drugs associated with ADRs differed substantially between studies due to differences between patient populations there were a number of similarities, and many of the drugs analysed in this review are commonly used in children. The results of this review will facilitate a greater understanding of prescribing practices, thus ultimately reduce drug harm. This may help in the development of interventions to improve drug prescribing and monitoring.

We examined the methods used for detecting, and assessing the causality, severity and avoidability of an ADR. The assessment of causality in individual cases of ADRs is required to establish whether there is an association between the untoward clinical event and the suspected drug [6]. The detection of ADRs depends on the validity and reliability of the tests employed and if sensitive methods are performed, in theory, all ADRs should be detected. We found a third (31/102) of studies did not report which causality assessment they used, with an additional six not using a recognised algorithm. As a consequence there may be either an underestimation or over estimation of ADRs in these studies. Over a third of studies (34/102) assessed ADRs for the severity of the reactions; just eight of which did not report any severe ADRs. Severe ADRs were described as those that caused either death or were directly life-threatening, caused hospital admission, prolonged hospitalisation or caused transfer to higher level of clinical care [113]. The ability to classify ADRs by severity provides a mechanism for clinicians to identify problem areas and implement interventions to inform paediatric pharmacovigilance practice.

The absence of avoidability data was most noticeable in this review; with only fourteen studies (14/102; 14%) providing avoidability data. Therefore it is not possible to consider this important aspect of drug safety in order to prevent future ADRs [7]. Further studies are clearly required to determine which ADRs are potentially avoidable. These studies could provide the necessary data in order to enable clinicians to administer medications in the safest and appropriate way.

The reporting quality of some of the included studies was poor, which may have affected the results. Not all provided a clear definition of the term ‘adverse drug reaction’; often insufficient information was in the publication in order to determine whether ADRs included medication or prescribing errors. ADR incidence data were not always clearly described in the publications. In many studies (n = 48/102) reporting was unclear regarding whether the incidence rate was reported at the patient and/or episode level and whether or not all children had been exposed to a drug.

It is disappointing given the large number of studies we identified which addressed this problem that most did not include these important methodological aspects. We recommend researchers should consider the approach which we have taken to assess the quality of these studies, although we recognise that further work is needed to develop a quality assessment tool which meets rigorous standards of development. We recommend that future studies provide information on the avoidability of ADRs; this may help in the development of interventions to improve drug prescribing and monitoring. There are several outcomes that warrant further investigation or require more detailed information to be collected. Important risk factor data and the number of medications each child received needs to be reported fully in order to explore possible sources of heterogeneity between studies. Future studies need to use clear, unambiguous terminology to describe how ADR incidence rates are calculated. This would improve understanding of the clinical relevance of individual study findings and allow comparisons between studies for the purposes of systematic review, enabling more robust conclusions and recommendations.

This review confirms previous studies which have shown ADRs to be an significant problem in children and has highlighted therapeutic classes of drugs most commonly associated with them. We strongly recommend further work to address prescribing practices in different settings and avoidability of ADRs is needed to indicate how such ADRs may be prevented.

## Supporting Information

Figure S1 **Flow diagram.**
(TIFF)

Checklist S1 **PRISMA Checklist.**
(DOC)
