# Supplementary figures and images for: Adverse Drug Reactions in Children—A Systematic Review
Source: PLoS One. 2012 Mar 5;7(3):e24061. doi: 10.1371/journal.pone.0024061 (PMC3293884; doi:10.1371/journal.pone.0024061)

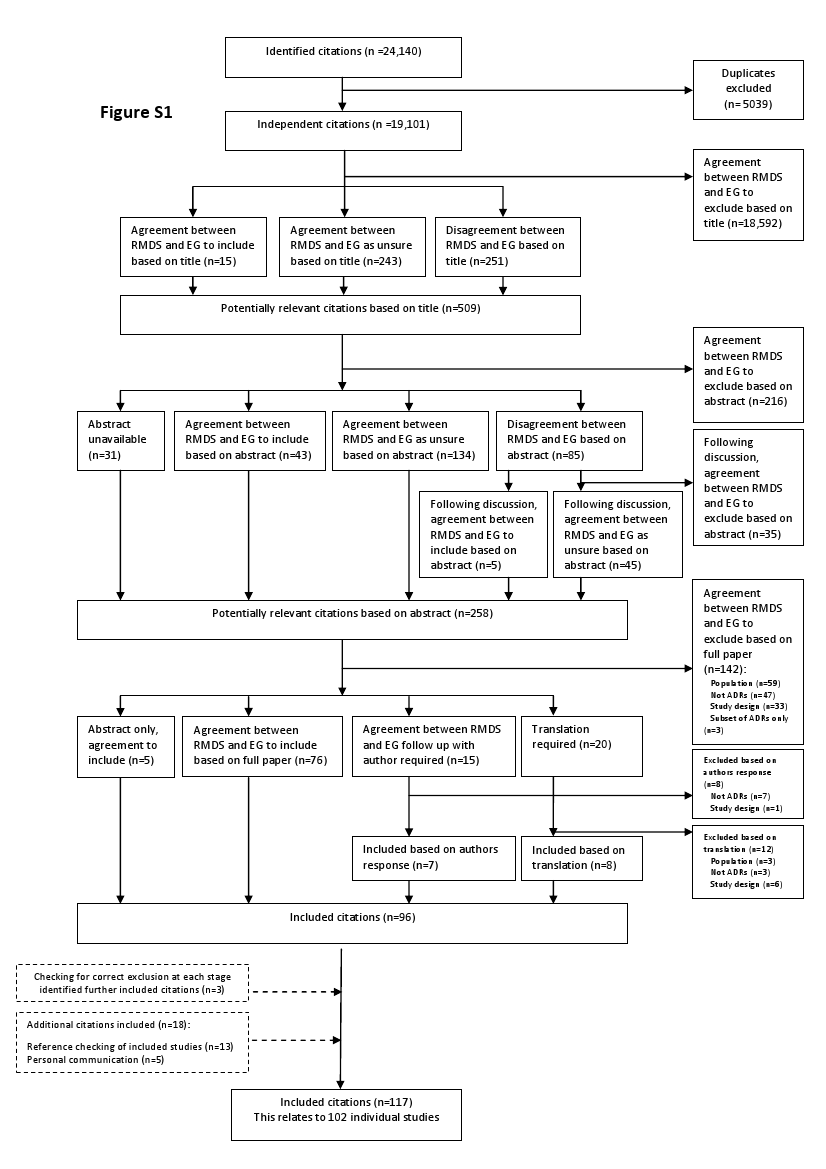

Supplement: Figure S1 — Flow diagram. (TIFF) [file pone.0024061.s001.tif]
